# Taurine attenuates *Listeria monocytogenes*-induced inflammation and pyroptosis in mouse model by regulating MAPK and NLRP3/caspase-1/GSDMD pathways

**DOI:** 10.1128/msystems.01043-25

**Published:** 2026-02-02

**Authors:** Tianqi Liu, Xiaoqing Zhang, Zanmei Qi, Xiaojiao Zheng, Yang Weng, Xue Han

**Affiliations:** 1 Department of Gastroenterology, First Affiliated Hospital of China Medical University 159407, Shenyang, Liaoning, China; 2 Department of Medical Basic Experimental Teaching Center, China Medical University 26488, Shenyang, Liaoning, China; 3 Department of Immunology, College of Basic Medical Sciences, China Medical University 599277 , Shenyang, Liaoning, China; 4 Department of Digestive Endoscopy, Fourth Affiliated Hospital of China Medical University 462540, Shenyang, Liaoning, China; University of South Florida Morsani College of Medicine, Tampa, Florida, USA

**Keywords:** taurine, *Listeria monocytogenes*, inflammation, pyroptosis, NLRP3

## Abstract

*Listeria monocytogenes*, as a significant foodborne pathogen, is not frequently encountered; however, when infections do occur, they can prove highly lethal to specific populations. Antibiotics are still regarded as the primary treatment option for *Listeria* infections. Nevertheless, under the global antibiotic crisis, there is an urgent demand for innovative and alternative strategies. In our study, we identified taurine, a sulfur-containing free amino acid that can be extracted from a wide variety of foods, as an effective inhibitor of *Listeria* growth. Furthermore, our findings revealed that taurine administration significantly reduced bacterial burden and concurrently mitigated host-derived inflammation in the mouse model. It was observed that taurine stimulated T-cell proliferation and inhibited pyroptosis via mitogen-activated protein kinase and NLRP3/caspase-1/GSDMD pathways. Our research outcomes position taurine as a promising therapeutic candidate for combating *Listeria* infections, with an inherent advantage of reduced likelihood for inducing antibiotic resistance compared to conventional antibiotic treatments.

**IMPORTANCE:**

*Listeria monocytogenes* infections are lethal to specific groups. With the antibiotic crisis, new treatments are needed. Taurine, a safe dietary compound, was found to inhibit *Listeria* growth. It targets both *L. monocytogenes* virulence and host immunopathology, stimulated T-cell proliferation, and inhibited pyroptosis. We establish taurine as the non-antibiotic agent that decouples bacterial cytotoxicity from inflammation-driven tissue damage, offering an immediately translatable strategy for high-risk infections amid the antibiotic resistance crisis.

## INTRODUCTION

*Listeria monocytogenes* (*Lm*), a gram-positive foodborne bacterium, is responsible for listeriosis and can be transmitted through various food items, including raw milk, dairy products, meat, and fresh produce. It can also persist in food processing environments. After consuming contaminated food, *Lm* can settle in the gastrointestinal tract and cause symptoms such as fever and diarrhea (1). Although the incidence of human listeriosis is low, its mortality rate can reach 20%–30% among susceptible populations, including immunocompromised individuals, neonates, pregnant women, and the elderly. These populations may experience severe complications like meningitis, liver abscess, and abortion. *Lm* infection thus poses a serious threat to public health and food safety (2). To date, antibiotics remain the primary treatment option for listeriosis. Similar to other bacteria, *Lm* treatment faces the challenge of antibiotic resistance, posing a serious threat to global public health (3). Therefore, the development of additional alternative or complementary therapies is an urgent necessity.

Taurine, a sulfur-containing amino acid widely distributed in the human body, plays a variety of physiological roles, including osmoregulation, bile acid conjugation, ion movement modulation, and antioxidation (4). Taurine is indeed found in various food sources. It is naturally present in animal-based products, particularly in high concentrations in seafood, organ meats, and dairy products (5). The consumption of taurine through these natural food sources suggests the overall safety of consuming this substance. Taurine, lauded for its health advantages, is a popular addition to both human foods and animal feeds. It is prevalent in functional beverages, supplements, and fortified edibles aimed at boosting exercise capacity, immunity, cardiovascular wellness, and neurological development. In animal nutrition, amid growing antibiotic restrictions, taurine emerges as a favored natural additive, enhancing immunity, disease resistance, growth, and reproductive capabilities in animals (6). Many studies have highlighted the crucial role of taurine in inflammation-related diseases. It has been observed that taurine and its derivatives possess therapeutic potential in managing various inflammatory disorders such as acne vulgaris, external otitis, purulently coated crural ulcerations, and keratoconjunctivitis (7–9). Taurine was found to alleviate pyroptotic cell death dependent upon the activation of NLRP3 inflammasome in As_2_O_3_-induced non-alcoholic steatohepatitis (NASH) (10), and it has also been proven that taurine can alleviate schistosoma-induced liver injury by inhibiting the TXNIP/NLRP3 inflammasome signal pathway and pyroptosis (11). Considering the regulatory effect of taurine on NLRP3 inflammasome activation and its anti-inflammatory properties, we hypothesized that it could also be a potential therapeutic approach for *Lm* infection. Thus, our study aims to elucidate the therapeutic potential of taurine in managing *Lm* infections by investigating its effects on bacterial growth, host inflammation, and immune responses. In particular, we examine the function of taurine in regulating the mitogen-activated protein kinase (MAPK) and NLRP3/caspase-1/GSDMD pathways. These pathways are essential for *Lm*-induced inflammation and pyroptosis. Our findings deepen our comprehension of the complex pathogenic mechanisms of *Lm* infections and offer fresh insights into the mechanisms of taurine’s protective effects. Additionally, it presents a potential alternative to conventional antibiotics, addressing the rising challenge of antimicrobial resistance.

## MATERIALS AND METHODS

### Bacterial strain and materials

The wild-type *L. monocytogenes* strain 10403S was stored in −80°C in our laboratory. It was passaged through BALB/c mice before preparation for stocks. *Lm* stocks were titrated and kept in 1 mL tryptic soy broth (TSB; Hopebio, HB4114)-glycerol at −80°C (12). Taurine (≥99%, Reagent grade) was purchased from Beyotime Biotechnology (Beijing, China).

### Determination of minimum inhibitory concentration and minimum bactericidal concentration of taurine against *Lm*

The minimum inhibitory concentration (MIC) and minimum bactericidal concentration (MBC) of taurine against *Lm* were determined using the broth microdilution method in accordance with guidelines from the Clinical and Laboratory Standards Institute (CLSI M07-A8). Briefly, serial twofold dilutions of taurine (ranging from 1,600 to 3.125 mM) were prepared and added into a 96-well plate with 100 μL in each row. A mid-logarithmic phase bacterial culture with a standard concentration of half McFarland (10^8^ colony-forming unit [CFU]/mL) was prepared, followed by a 1:100 dilution in TSB to achieve a working inoculum of approximately 10^6^ CFU/mL and 100 μL was added to the wells. The MIC was recorded as the lowest concentration that completely inhibited visible growth after 18–24 h of incubation at 37°C. To evaluate the MBC of the study groups, aliquots of 50 μL from all the wells which showed no visible bacterial growth were seeded on the solid culture (tryptic soy agar [TSA]; Hopebio, HB0177) and were determined after 24 h at 37°C based on observation of microbial colony formation or non-formation.

### Growth curves and CFU determination

The growth curve of *Lm* was measured to detect the antibacterial effect of taurine according to the method previously described (13, 14). *Lm* was cultured in TSB at 37°C with shaking at 200 rpm for 16 h, then 1 mL overnight culture was added into 100 mL TSB containing indicated concentrations of taurine (80, 400, and 800 mM), and a corresponding unit conversion table is provided (Table S1) for clarity. The bacteria culture without taurine was used as the control. To control for the effects of increased osmolarity, an equiosmolar control (800 mM mannitol) was included in the bacterial growth assays. All samples were incubated at 37°C and 200 rpm, and the absorption values at 600 nm were determined at 1, 2, 4, 6, 8, 10, and 12 h. The growth curves were generated by plotting the optical density at 600 nm (OD_600_) values versus time. The number of CFU was calculated by plating serial dilutions of the bacteria at different time points on TSA plates. After incubation at 37°C for 24 h, the number of bacteria was counted. The experiment was performed in triplicate.

### Hemolysis and biofilm formation assay

To evaluate the ability of taurine to inhibit hemolysis, hemolytic activity of the *Lm* culture supernatant was measured as described previously with minor modifications (13, 14). Overnight cultures of *Lm* were transferred into TSB (1:100) with different concentrations of taurine (80, 400, and 800 mM), along with an equiosmolar control of 800 mM mannitol. After incubation for 16 h at 37°C, following centrifugation (10,000 rpm, 5 min), 100 μL of the supernatant from each co-culture sample was incubated with sheep erythrocytes (25 μL) and PBS (875 μL) for 3 h. The system was centrifuged for 5 min at 10,000 rpm, and the absorption value at OD at 543 nm of the supernatant was detected to determine the hemolytic activity. The activity of each sample was compared with that of the positive control sample (sheep erythrocytes added to PBS supplemented with 2% Triton X-100), which was set as 100%. Concurrently, the bacterial density (OD_600_) of the culture was measured, and the hemolytic activity was normalized to this value. A previously reported crystal violet staining method was used to assess the effect of taurine on the amount of biofilm formation in *Lm*; 100 μL of bacterial suspension (1 × 10^8^ CFU/mL) was inoculated into 96-well plates containing different concentrations of taurine (80, 400, and 800 mM) incubated at 37°C for 24 h. After incubation, the supernatant was discarded; the bacteria were washed three times with PBS; and the OD_600_ of the adhered biofilm was measured for normalization. Then, the adherent biofilm was adsorbed with 200 µL of methanol for 15 min. After air-drying, the samples in each well were stained with 1% crystal violet solution for 5 min, and the excess crystal violet was washed with PBS and removed. The 96-well plates were dried at 60°C for 10 min, and then the absorbance at 595 nm was measured after dissolving the samples with 100 µL 95% ethanol (15). The final biofilm formation was expressed as the ratio of OD_595_ to OD_600_.

### Transcriptome sequencing and sequence data analysis

To investigate the impact of taurine on *Lm* at the transcriptional level, we established a treatment group that received 800 mM taurine and a control group without taurine, both grown in TSB culture media, with three biological replicates for each group. *Lm* was incubated at 37°C for 6 h, followed by centrifugation at 12,000 × *g* for 20 min at 4°C to collect the bacterial cells for transcriptome sequencing. Total RNA was extracted using TRIzol reagent (Invitrogen Life Technologies, USA), and mRNA was purified from the total RNA before synthesizing cDNA with random hexamer primers and M-MuLV Reverse Transcriptase. To isolate cDNA fragments with a preferred length of 370–420 bp, the library fragments were purified using the AMPure XP system (Beckman Coulter, Beverly, USA), and the quality of the library was assessed on the Agilent Bioanalyzer 2100 System. Ultimately, the cDNAs were sequenced on an Illumina platform, following the manufacturer’s protocols.

Clean data (clean reads) were generated by filtering out reads containing adapter sequences, reads with N bases, and low-quality reads from the raw data. The remaining clean reads were subsequently mapped to the reference genome using Bowtie2 (version 2.3.2). Read counts for each gene were determined using HTSeq (version 0.6.1). Differential expression analysis between the two conditions (with three biological replicates for each group) was conducted using the DESeq R package (version 1.18.0). *P* values were adjusted, applying the Benjamini and Hochberg method, with a corrected *P* value threshold of 0.005 and a log2 (fold change) of 1 set for identifying significantly differentially expressed genes. Gene Ontology (GO) enrichment analysis for the differentially expressed genes was performed using the GOseq R package, and the KOBAS software was utilized to assess the statistical enrichment of these differential expression genes in Kyoto Encyclopedia of Genes and Genomes (KEGG) pathways.

### Cell line infection

Mouse J774.1 macrophage-like cells were grown in high-glucose DMEM plus 10% fetal calf serum (FCS), penicillin (100 U/mL), and streptomycin (100 U/mL) (13). The cells were plated in 96-well plates (approximately 2 × 10^4^ cells/well) and cultured overnight, then incubated with taurine at concentrations ranging from 12.5 to 400 mM for 24 h. Cell viability was determined by using a Cell Counting Kit (CCK8; EallBio Life Sciences, Beijing, China). After another incubation period at 37°C/5% CO_2_ for 2–4 h, the OD at 450 nm of each sample was obtained with a microplate reader. Cell viability was expressed as a percentage relative to cells without taurine treatment following the removal of background value (CCK8 + DMEM).

For the *in vitro* experimental infections with *Lm*, J774.1 was cultured in a 96-well plate as described above (16). For the assessment of intracellular bacteria, cells were incubated with overnight culture *Lm* at 37°C for 4 h at a multiplicity of infection (MOI) of 10. The supernatant was discarded, and then the cells were washed with sterile PBS three times, and gentamicin (100 µg/mL) was added for 1 h to kill the residual extracellular bacteria. Afterward, the cells were lysed with 0.2% Triton X-100, and the colony count was determined by serial dilutions and drop plate method on TSA plates after overnight incubation at 37°C.

### Cytotoxicity assays

Cell death was assessed using a lactate dehydrogenase (LDH) release assay kit (Beyotime Biotechnology). To evaluate *Lm*-induced cytotoxicity, J774.1 grown overnight in the 96-well plate at 2 × 10^4^ cells/well were challenged with *Lm* at an MOI of 10. Meanwhile, the indicated concentration of taurine was added into the culture system. To inhibit NLRP3, cells were pretreated with MCC950 (10 μM) for 2 h prior to infection. Following a 4h incubation at 37°C, cytotoxicity was quantified by measuring the LDH released into the supernatant following the manufacturer’s protocol (16). Culture medium alone and medium containing 0.5% Triton X-100 served as negative and positive controls, respectively. Apoptosis was further analyzed using an apoptosis detection kit (Beyotime Biotechnology), where fluorochrome FITC-labeled annexin V was employed to specifically label and detect apoptotic cells. Stained cells were then examined by flow cytometry to ascertain the percentage of apoptotic cells among the treated population (16).

### Animal experiment

The BALB/c mouse strain was chosen as the infection model as it is susceptible to *Lm* infection, making it a suitable model for studying the pathogenesis and treatment of *Lm* infection, which closely mimics human disease. Female BALB/c mice aged 6–8 weeks, weighing between 18 and 20 g, were purchased from SPF Biotechnology Co. Ltd. (Beijing, China) and maintained at the Animal Care Facilities of the China Medical University. The mice were housed under standard light and temperature conditions and provided with unlimited access to water and food.

Before infection, *Lm* stock was thawed on ice and incubated in 9 mL of TSB for 1 h at 37°C, washed, and resuspended to the desired titer in PBS. The mice were injected intravenously (i.v.) with 2×10^4^ CFU live *Lm* in 400 µL PBS via tail vein (17). Taurine (100, 200, 400, and 800 mg/kg) was administered intraperitoneally at 2 h post-infection and daily for 3 days. The mice injected with PBS were assigned as control. In the inhibitor treatment groups, the p38 MAPK inhibitor SB203580 (5 mg/kg/day) was administered intraperitoneally starting from the day of infection for three consecutive days. The mice (*n* = 10 per group) were infected with *Lm* and monitored for survival and body weight until day 9. For other analyses, a separate cohort of mice (*n* = 6 per group) was infected and euthanized at 72 h post-infection to collect tissues. The mice were infected and randomly assigned to each group.

The liver, spleen, and brain were collected and homogenized in sterile PBS to obtain the bacterial burden (18). Then, the activity of myeloperoxidase (MPO) and hydrogen peroxide (H_2_O_2_) content in fresh liver tissue was detected according to the manufacturer’s instructions (Jiancheng, Nanjing, China) (19). Organ tissues were simultaneously fixed in 10% buffered formalin and embedded in paraffin for histological assessment. Hematoxylin and eosin staining was examined by light microscopy for histological tissue damage evaluation. Terminal deoxynucleotidyl transferase dUTP nick end labeling (TUNEL) staining was performed according to the manufacturers’ protocol. The immunofluorescent staining of CD4, CD8, ASC, and caspase-1 was also performed. The images were observed and photographed with a fluorescent microscope. All sections were evaluated in three consecutive microscopic fields for assessment.

For the test of the permeability of the blood-brain barrier, after 72 h of infection, the mice were injected i.v. with 200 µL 2% Evans Blue solution. One hour later, the mice were anesthetized and perfused intracardially with 30 mL PBS and then sacrificed. The brain coloration was assessed and incubated in 2 mL formamide for 48 h at 37°C. After 2 days of formamide extraction, Evans Blue absorption was determined in 100 µL formamide extracts by measuring with the microplate reader at 630 nm (12).

### Pharmacokinetics study

To investigate the *in vivo* distribution and kinetics of taurine, female BALB/c mice (6–8 weeks old) received a single intraperitoneal injection of taurine (400 mg/kg) (*n* = 3 per time point). At predetermined time points post-administration (0, 0.5, 1.0, 2.0, 4.0, 6.0, 12.0, 24.0, 48.0, and 72.0 h), blood samples were collected and centrifuged to obtain serum. Key organs, including the liver and spleen, were harvested. The concentrations of taurine in serum and tissue homogenates were quantified using a commercial taurine assay kit (MET-5071; Cell Biolabs, San Diego, CA, USA) according to the manufacturer’s instructions. The tissue concentrations were normalized to the weight of the tissue sample and expressed as micromolar per gram. The mean concentration at each time point was used to plot the concentration-time curve.

### Splenocyte isolation and flow cytometry

Splenocytes were harvested as previously described. The spleen was removed at 72 h after infection, and a single-cell suspension was obtained by grinding the tissue through a stainless steel mesh. Erythrocytes were lysed with cold 0.17 M NH_4_Cl, and the cells were washed twice with PBS. Viability was determined by the cell ability to exclude Trypan blue dye. Spleen cells were adjusted to a final concentration of 10^7^ cells/mL in RPMI 1640 supplemented with 10% FCS.

For surface antibody staining, splenocytes were washed twice with PBS supplemented with 2% FCS (FACS buffer). Each sample containing 10^6^ cells was first blocked with anti-mouse CD16/CD32 (FcIII/II receptor) antibody and then stained with FITC-conjugated anti-CD4 (GK1.5) and PE-conjugated anti-CD8a (53.0–6.7) antibodies. For intracellular staining, freshly isolated splenocytes were stimulated with PMA (50 ng/mL) and ionomycin (1 µg/mL) for 6 h in the presence of Golgi blocker monensin (2 µM). After surface staining, the cells were then fixed, permeabilized, and stained with APC-conjugated anti-IFN-γ (XMG1.2) or isotype control rat IgG1 (R3-34) mAbs. Finally, stained cells were fixed with 1% paraformaldehyde and acquired using a NovoCyte flow cytometer (Agilent, USA). All data were analyzed with NovoExpress software 1.6.2 (Agilent).

### RNA isolation and real-time PCR

Total RNA was isolated from mouse spleen 72 h after infection. Samples were homogenized in 1 mL of TRIzol (Invitrogen Life Technologies) and reverse transcribed with HiScript III 1st Strand cDNA Synthesis Kit (Vazyme, Nanjing, China, R323). The levels of IL-6, IL-1β, iNOS, and TNF-α mRNA were measured by real-time PCR using ChamQ SYBR Color qPCR Master Mix (Vazyme, Q311). Bacterial RNA extraction was performed according to the manufacturer’s protocol (MolPure Bacterial RNA Kit; Yeasen, China), with gene expression normalized to 16S rRNA. The relative expression of the target genes was calculated by the ΔΔCt method, and each sample was done in triplicate. The primer sequences for PCR amplification are shown in Table S2 (12).

### Immunoblot analysis

Protein electrophoresis samples were prepared by taking liver tissues from each group of mice, extracting proteins using RIPA lysate containing protease inhibitors and phosphatase inhibitors. Protein concentrations were determined using a BCA Protein assay kit (Beyotime Biotechnology). Equal amounts of protein (30 μg per lane) were separated by 10% SDS-PAGE gel electrophoresis and transferred to PVDF membrane with 5% skimmed milk closed for 1 h. The target proteins on the membranes were probed with the specific primary antibodies as follows: rabbit anti-JNK1 + JNK2 + JNK3 polyclonal antibody (Bioss, bs-2592R), rabbit anti-phospho-JNK1/2/3 (T183 + T183 + T221) monoclonal antibody (Bioss, bsm-52462R), rabbit anti-ERK1/2 monoclonal antibody (Bioss, bsm-52259R), rabbit anti-phospho-ERK1/2 (Thr202 + Thr204) polyclonal antibody (Bioss, bs-3016R), p38 MAPK rabbit mAb (CST, 8690), phospho-p38 MAPK (Thr180/Tyr182) rabbit mAb (CST, 4511), IL-1β pAb (Bioss, bs-812R), caspase 1/p20/p10 polyclonal antibody (Proteintech, 22915-1-AP), total and cleaved N-terminal GSDMD antibody (Abmart, P79887R), and NLRP3 monoclonal antibody (Proteintech, 68102-1-Ig). These primary antibodies were diluted at a 1:1,000 dilution in 5% BSA/TBST. The reference antibody β-actin (Servicebio, GB15003) or GAPDH (Servicebio, GB11002) was diluted to 1:5,000 for use. After incubation overnight at 4°C, the corresponding secondary antibody HRP goat anti-mouse IgG (ABclonal, AS003) and HRP-conjugated goat anti-rabbit IgG (ABclonal, AS014) were applied, with a dilution ratio of 1:20,000. The immunoblots were developed with the Enhanced Chemiluminescence ECL Kit (Tanon, 180-501) and visualized using a Western blotting detection system (Tanon, China). The protein bands were analyzed using ImageJ software (12). Each experimental group included three biological replicates per lane. Full uncropped gel images with protein markers are provided in Fig. S6.

### Statistical analysis

Data are expressed as mean with SD from at least three independent experiments performed in triplicate. The data were analyzed using SPSS 19.0 (IBM, USA). Statistical significance of differences between the two groups was determined using Student’s *t*-test; for multiple groups, one-way ANOVA followed by Tukey’s *post hoc* test was used. The statistical significance of the difference in survival rate was analyzed by the log-rank test. Differences were considered significant when *P* < 0.05.

## RESULTS

### Taurine inhibits *Lm* growth, hemolytic activity, and biofilm formation

**Fig 1:**
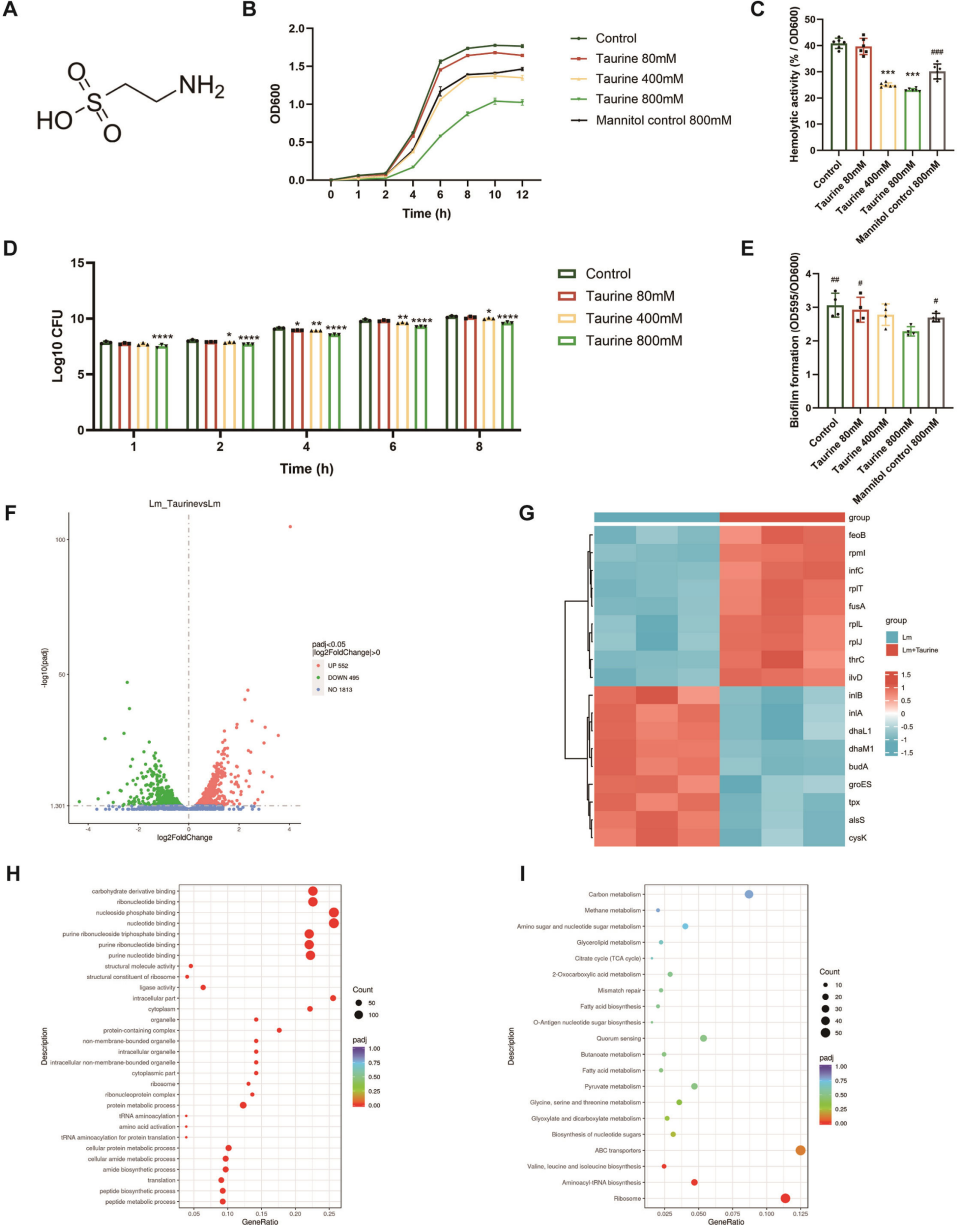
Taurine inhibits *Lm* growth, hemolytic activity, and biofilm formation. (**A**) The chemical structure of taurine. (**B**) Growth kinetics curve of *Lm* treated with different concentrations of taurine. (**C**) Hemolytic activity of *Lm* culture supernatants with different concentrations of taurine (*n* = 3 biological replicates). (**D**) The number of colony-forming units (CFU) was calculated at different time points on TSA plates (*n* = 3 biological replicates). (**E**) Biofilm assay by microtiter plate with crystal violet staining (*n* = 4 biological replicates). (**F**) Volcano plot of the number of differentially expressed genes (DEGs) between *Lm* and *Lm* + taurine groups. (**G**) DEG heat map between *Lm* and *Lm* + taurine groups. (**H**) GO enrichment analysis of DEGs. (**I**) KEGG pathway enrichment analysis of DEGs. Data are expressed as mean ± SD. Statistical significance was determined by one-way ANOVA with Tukey’s *post hoc* test. The symbol * denotes comparison to the control group (*P < 0.05, **P < 0.01, ***P < 0.001, ****P < 0.0001). The symbol # denotes comparison to the taurine 800 mM group (^#^*P* < 0.05, ^##^*P* < 0.01, ^###^*P* < 0.001).

As reported in a previous study, taurine is an amino acid derivative, which is composed of a sulfonic acid group attached to the beta-carbon of an amino acid (20). The structure of taurine is shown in Fig. 1A. To benchmark the direct antibacterial efficacy of taurine, we first determined its MIC and MBC using the standard broth microdilution assay. The MIC was found to be 100 mM, indicating that this concentration is sufficient to suppress visible bacterial growth. However, subculturing assays revealed that taurine lacked bactericidal activity even at the highest concentration tested (1,600 mM), with an MBC exceeding this value. The resulting MBC/MIC ratio of >16 definitively classifies taurine as a bacteriostatic agent against *Lm* (Fig. S1). Taurine demonstrated a concentration-dependent inhibitory effect on *Lm* growth. At the highest concentration tested (800 mM), taurine delayed the growth plateau by 2 h and reduced CFU by 50% compared to the control group (Fig. 1B and D). Importantly, the inclusion of an 800 mM mannitol control, which exhibited a growth profile distinct from 800 mM taurine, confirmed that the observed inhibition is a specific biological effect rather than a consequence of osmolarity. In addition to inhibiting growth, taurine significantly reduced the virulence of *Lm*. Critically, the specific hemolytic activity (normalized to bacterial density) was reduced by nearly half at 800 mM, an effect significantly greater than that of an equiosmolar control, indicating a specific biological inhibition beyond osmolarity. Furthermore, the biofilm formation was mitigated by 24% at a concentration of 800 mM (Fig. 1C and E). These results suggest that taurine can inhibit bacterial replication and impair important pathogenic processes.

### Taurine may inhibit the growth of *Lm* by affecting its metabolic pathways

We selected a concentration of 800 mM of taurine for performing bacterial transcriptome sequencing. All RNA-seq samples passed quality control (Table S3) and were subsequently analyzed for differential gene expression. As shown in Fig. 1F through I, transcriptomic analyses revealed that treatment with taurine significantly altered the gene expression profile of *Lm*. For instance, 552 genes were upregulated, while 495 genes were downregulated. To better understand the biological functional categories of DEGs, we annotated and analyzed the DEGs using the GO database to categorize gene functions and descriptions. A total of 51 functional groups were annotated to the DEGs, with 11 cellular processes, 16 biological processes (BPs), and 24 molecular functions (MFs) changed. We found that for the BP category, most DEGs were associated with protein metabolism process, amide metabolism process, and peptide metabolism process. As far as the cellular composition category is concerned, the GO functional components were dominated by intracellular components such as ribosomes, and in the MF category, DEGs were mainly associated with various types of nucleotide binding. KEGG pathway enrichment analysis underscored the significant involvement of these DEGs in metabolic pathways, specifically highlighting their role in ribosome, aminoacyl-tRNA biosynthesis, valine, leucine, and isoleucine biosynthesis. Functional enrichment analysis revealed that the implicated genes were primarily associated with ribosome biogenesis and amino acid metabolism. We experimentally verified the expression patterns of six candidate genes involved in virulence (inlA and inlB) and central metabolism (dhal_1, budA, alsS, and cysk) using qRT-PCR (Fig. S2). The result confirmed their significant downregulation in the taurine-treated group. This implies that taurine may suppress *Lm* growth by disrupting essential metabolic processes.

### Taurine can effectively alleviate *Lm*-mediated cytotoxicity and pyroptosis in J774.1 macrophages

**Fig 2:**
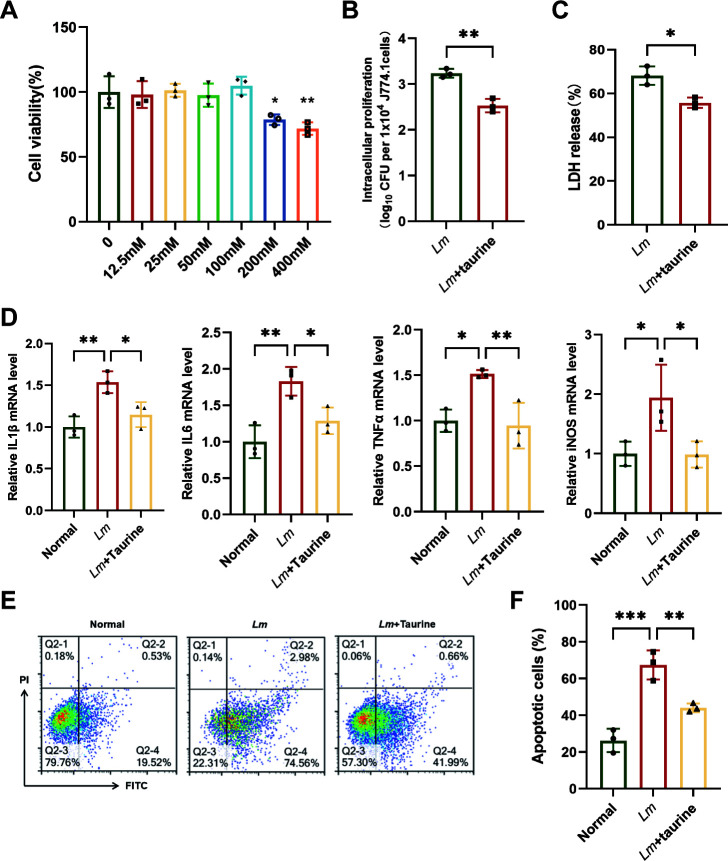
Taurine can effectively alleviate *Lm*-mediated cytotoxicity in J774.1 macrophages. (**A**) J774.1 cells were treated with indicated concentrations of taurine for 24 h, and cell viability was detected with the CCK-8 kit. (**B**) J774.1 cells were co-cultured with *Lm* in the presence or absence of taurine (100 mM), and the intracellular proliferation of bacteria was detected. (**C**) The level of LDH released into the supernatants of cells was detected using a cytotoxicity detection kit. (**D**) Levels of IL-1β, IL-6, TNF-α, and iNOS were detected by RT-qPCR. (**E**) Effect of taurine on apoptosis in J774.1 cells co-cultured with *Lm* as determined using flow cytometry. (**F**) The quantification analysis of apoptosis. Data are expressed as mean ± SD (*n* = 3 biological replicates). *P < 0.05, **P < 0.01, ***P < 0.001.

The efficacy of taurine in suppressing *Lm* growth and its hemolytic activity has been well established. To further explore the role of taurine in *Lm* infection *in vitro*, we detected bacteria growth in J774.1 macrophages with or without taurine administration. Prior cytotoxicity assessments with J774.1 cells (Fig. 2A) assured us of taurine’s non-toxic nature, leading us to select a concentration of 100 mM for subsequent investigations. Treatment with taurine (100 mM) prior to infection significantly reduced the survival of intracellular *Lm* by 79% and decreased LDH release by 12.4% in J774.1 macrophages (Fig. 2B and C). This suggests that taurine can protect cells from bacterial invasion and cytotoxicity. Furthermore, taurine suppressed the *Lm*-triggered increase in IL-1β, IL-6, TNF-α, and iNOS expression (Fig. 2D) and decreased apoptosis by 14.56% (Fig. 2E and F). To delineate the underlying mechanism, we investigated the NLRP3 inflammasome pathway, a key mediator of pyroptosis. Western blot analysis confirmed that taurine significantly suppressed *Lm*-induced activation of the NLRP3 inflammasome and IL-1β (Fig. S3A and B). We performed a rescue experiment using the NLRP3-specific inhibitor MCC950. Crucially, the ability of taurine to reduce the levels of IL-1β, caspase-1, and the N-terminal fragment of GSDMD, as well as to attenuate cell death, was abolished when NLRP3 was pharmacologically inhibited by MCC950 (Fig. S3C through F). Collectively, these findings indicate that taurine treatment could effectively restrain the replication of *Lm* in host cells and protect against *Lm*-induced cellular injury by specifically inhibiting the NLRP3 inflammasome pathway.

### Taurine improves survival and reduces bacterial burden in mice

**Fig 3:**
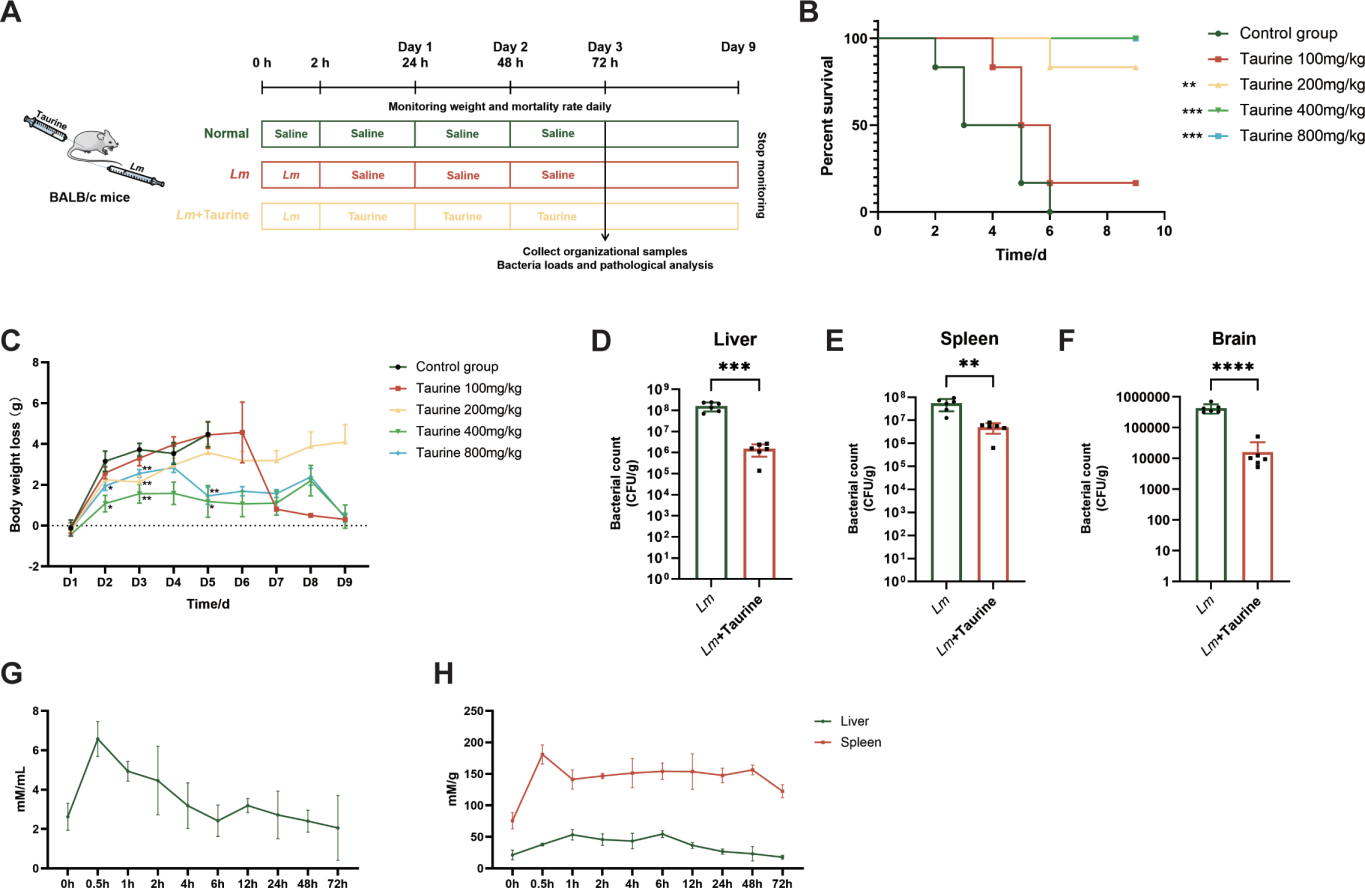
Taurine ameliorated the symptoms in an *Lm*-infected mouse model. (**A**) The experimental design. Mice were intraperitoneally injected with 2 × 10^4^ CFU live *Lm* and treated with indicated concentrations of taurine 2 h after *Lm* inoculation for 3 days. The survival rate (**B**) and weight loss (**C**) of the infected mice were observed for 9 days (*n* = 10 biological replicates per group). The number of colonies in the liver (**D**), spleen (**E**), and brain (**F**) was calculated (*n* = 6 biological replicates per group). (**G**) Concentration-time curve of taurine in serum after a single oral administration of 400 mg/kg (*n* = 3 biological replicates per group). (**H**) Concentration-time curves of taurine in the liver and spleen (*n* = 3 biological replicates per group). Data are expressed as mean ± SD. Significant difference from the control group was designated as *P < 0.05, **P < 0.01, ***P < 0.001, and ****P < 0.0001.

To examine the therapeutic effects of taurine on *Lm*-infected mice, BALB/c mice were intravenously challenged with 2 × 10^4^ CFU of *Lm* and then administered taurine at doses from 100 to 800 mg/kg (Fig. 3A). The results showed that taurine markedly enhanced survival rates in a dose-dependent manner. The dose of 400 mg/kg resulted in 100% survival, compared to the control controls, which had complete mortality by day 9 post-infection (Fig. 3B). Mouse body weight was also monitored during the entire experiment period (Fig. 3C), and the results exhibited that the taurine treatment group at the doses of 400 and 800 mg/kg experienced a slight weight loss from day 2, guiding the selection of 400 mg/kg as the optimal dose for further experiments. Furthermore, taurine treatment at a dose of 400 mg/kg significantly reduced bacterial colonization in critical organs. In particular, the liver showed a 59-fold decrease in CFU, the spleen a 6.8-fold decrease, and the brain a 20-fold decrease compared to infected controls (Fig. 3D through F).

### Taurine exhibits favorable pharmacokinetics with high accumulation in major target organs

To provide a pharmacokinetic basis for the observed protective effects of taurine *in vivo*, we measured its concentrations in serum, liver, and spleen following a single intraperitoneal dose of 400 mg/kg. As shown in Fig. 3G and H, taurine was rapidly absorbed into the bloodstream, reaching the maximum concentration (C~max~) of 6.6 mM at the time of maximum concentration (T~max~) of 0.5 h post administration, after which the serum concentration declined rapidly. Strikingly, taurine demonstrated extensive tissue distribution and remarkable retention in both the liver and spleen. The drug rapidly accumulated in these target organs, with the spleen achieving a remarkable C~max~ of 180 mM at 0.5 h. Simultaneously, the liver also showed robust uptake, reaching a C~max~ of 53.3 mM at a slightly later T~max~ of 1 h. Throughout the experimental period, the concentration of taurine in both organs was substantially higher than that in serum. Notably, the spleen displayed the most sustained drug exposure, forming a prolonged plateau above 140 mM for up to 48 h, while the liver also maintained significantly elevated levels. These results indicate that administered taurine efficiently targets and accumulates in the key organs of infection, achieving and maintaining high local concentrations for an extended duration.

### Taurine effectively attenuates *Lm* pathogenicity *in vivo*

**Fig 4:**
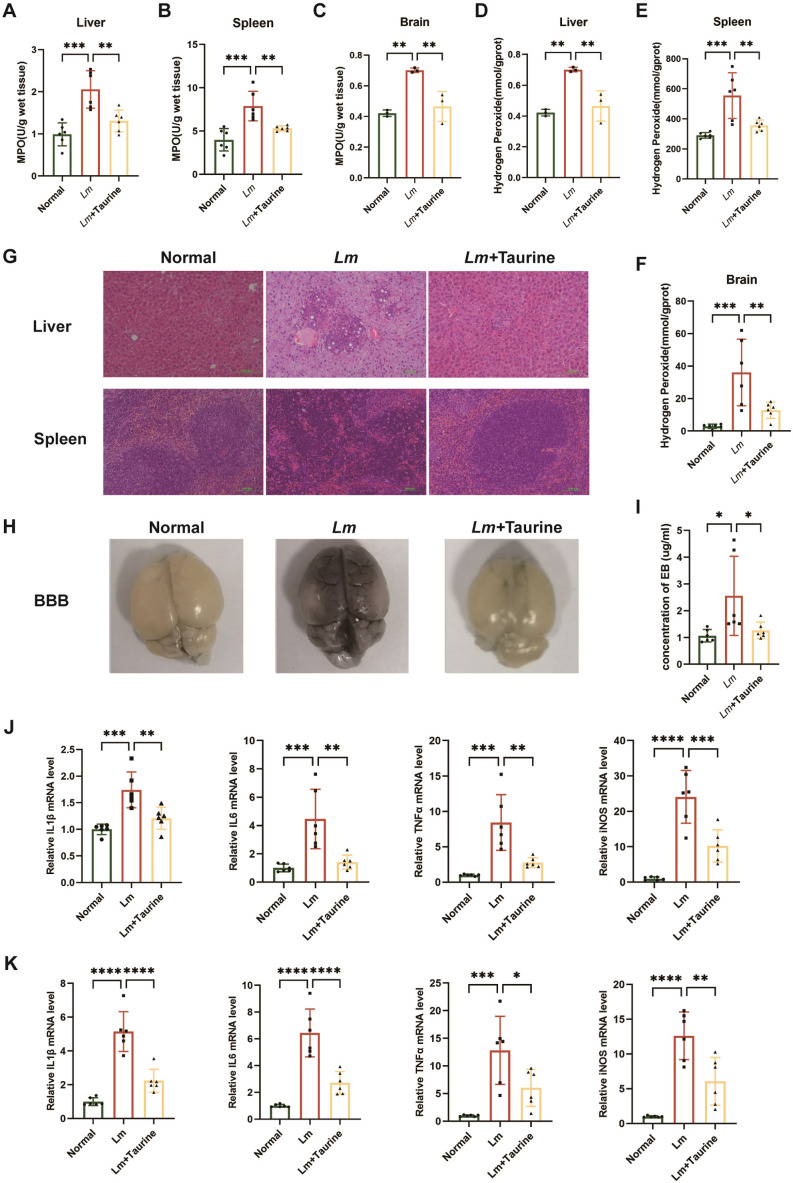
Taurine suppressed tissue damage and inflammatory cytokine expression in an *Lm*-infected mouse model. The MPO activities and H_2_O_2_ concentration of livers (**A and D**), spleens (**B and E**), and brains (**C and F**) are shown. Histopathological analyses of livers and spleens (**G**). The blood-brain barrier (BBB) integrity (**H**) and score (**I**). The mRNA levels of IL-1β, IL-6, TNF-α, and iNOS in the liver tissue (**J**) and spleen tissue (**K**) were detected by RT-qPCR (*n* = 6 biological replicates per group). Data are expressed as mean ± SD from three independent experiments. *P* values were determined by one-way ANOVA with Tukey’s test. *P < 0.05, **P < 0.01, ***P < 0.001, ****P < 0.0001.

To further assess the protective effects of taurine against *Lm* infection, the key markers of oxidative stress and tissue damage—MPO and H_2_O_2_ levels in liver, spleen, and brain—were evaluated. As presented in Fig. 4A through F, taurine effectively suppressed *Lm*-induced oxidative stress by reducing MPO activity and H_2_O_2_ levels. Histopathological assessments provided further evidence of the protective role of taurine. Mice infected with *Lm* showed severe hepatic necrosis and splenic germinal center destruction, which were significantly improved by taurine treatment (Fig. 4G). Furthermore, taurine improved the blood-brain barrier’s integrity, as indicated by a significant decrease in Evans Blue leakage (Fig. 4H and I). Altogether, taurine has a protective effect against *Lm* infection, efficaciously suppressing bacterial loads in vital organs and thereby demonstrating its substantial therapeutic potential. The process of inflammation is triggered when there is an infection threat to the host. As shown in Fig. 4J and K, consistent with the oxidative stress and pathological analysis, *Lm* infection significantly elevated the level of IL-1β, IL-6, TNF-α, and iNOS in liver and spleen tissues. In summary, these findings suggest that taurine exerts its therapeutic effects against *Lm* infection through two key mechanisms: direct inhibition of bacterial growth and resolution of host inflammatory responses.

### Taurine enhances T-cell immune responses against *Lm* infection

**Fig 5:**
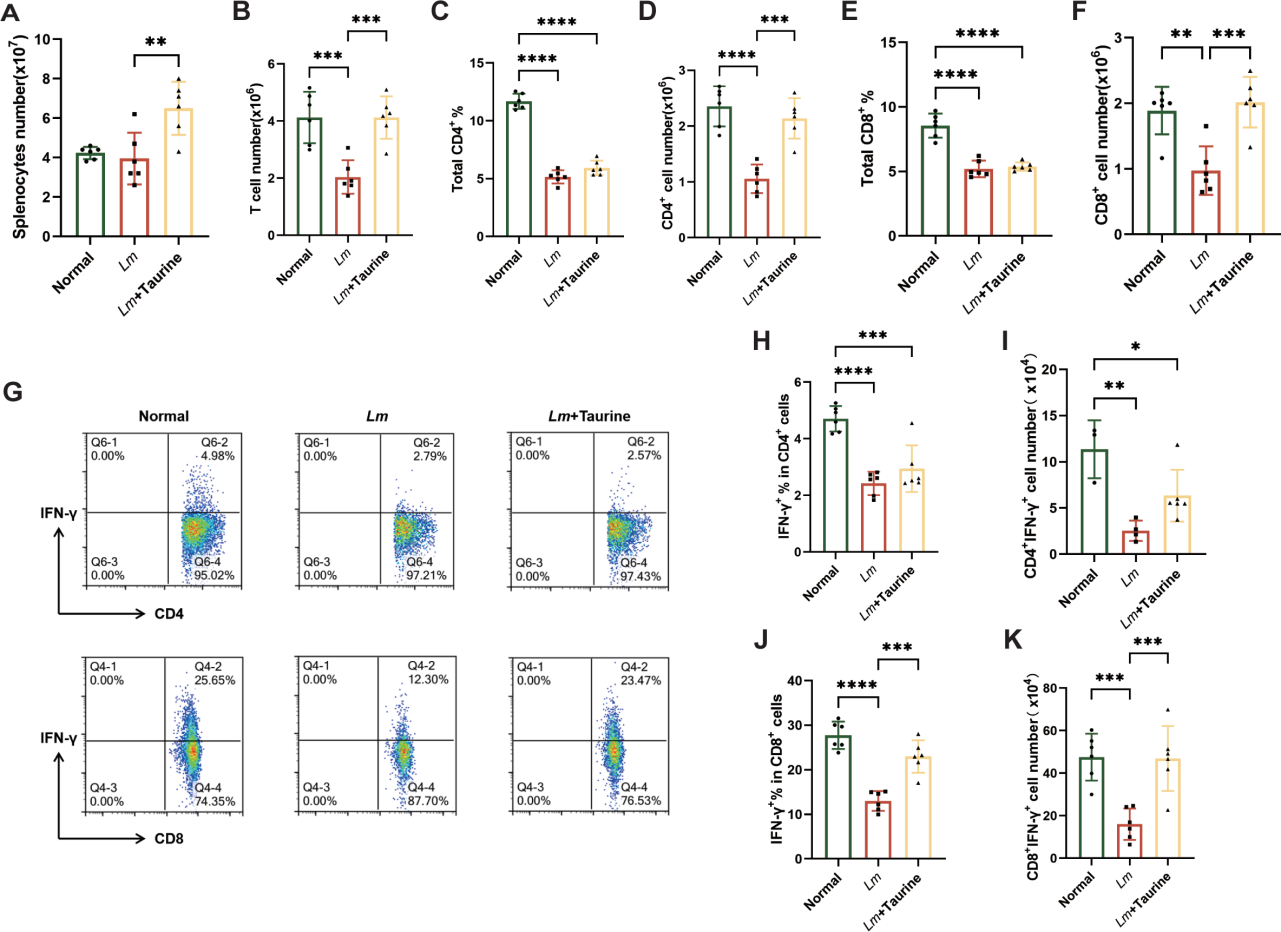
Flow cytometry was used to analyze the splenocytes. (**A**) Total number of splenocytes. (**B**) Total number of T cells. (**C**) The percentage of CD4^+^ T cells. (**D**) Total number of CD4^+^ T cells. (**E**) The percentage of CD8^+^ T cells. (**F**) Total number of CD8^+^ T cells. (**G**) Flow scatter plot of all test indicators. (**H**) The percentage of IFN-γ^+^ cells in the CD4^+^ subset. (**I**) The absolute number of IFN-γ^+^ CD4^+^ cells. (**J**) The percentage of IFN-γ^+^ cells in the CD8^+^ subset. (**K**) The absolute number of IFN-γ^+^ CD8^+^ cells (*n* = 6 biological replicates per group). Data are expressed as mean ± SD from three independent experiments. *P* values were determined by one-way ANOVA with Tukey’s test. *P < 0.05, **P < 0.01, ***P < 0.001, ****P < 0.0001.

To investigate the immunomodulatory effects of taurine, we examined splenic T-cell responses in mice infected with *Lm*. Mice treated with taurine showed a substantial recovery from *Lm*-induced splenocyte depletion, with a 2.5-fold increase in total splenocyte numbers compared to untreated infected mice (Fig. 5A). Flow cytometry analysis showed that taurine effectively countered the infection-driven decrease in T-cell counts, increasing their absolute numbers from 1.2 × 10⁶ to 3.8 × 10⁶ per spleen (Fig. 5B). Although the percentage of splenic CD4^+^ and CD8^+^ T cells did not differ between the *Lm* infection group and the taurine treatment group, the absolute numbers of CD4^+^ and CD8^+^ T cells in the taurine treatment group basically restored to normal levels (Fig. 5C through F). The expression of IFN-γ inside T cells was also detected (Fig. 5G). Two infected groups exhibited a significantly decreased percentage of IFN-γ^+^ CD4^+^ in comparison with the control group (Fig. 5H). The total number of IFN-γ^+^ CD4^+^ cells in the taurine treatment group changed slightly, but no significant difference was observed (Fig. 5I). Besides, there was a remarkably higher proportion of IFN-γ^+^ CD8^+^ in the taurine treatment group than those in the *Lm*-mono-infected group, including the absolute number of IFN-γ^+^ CD8^+^ cells (Fig. 5J and K).

### Taurine enhances MAPK signaling pathway and downregulates *Lm*-activated NLRP3 inflammasome in mice

**Fig 6:**
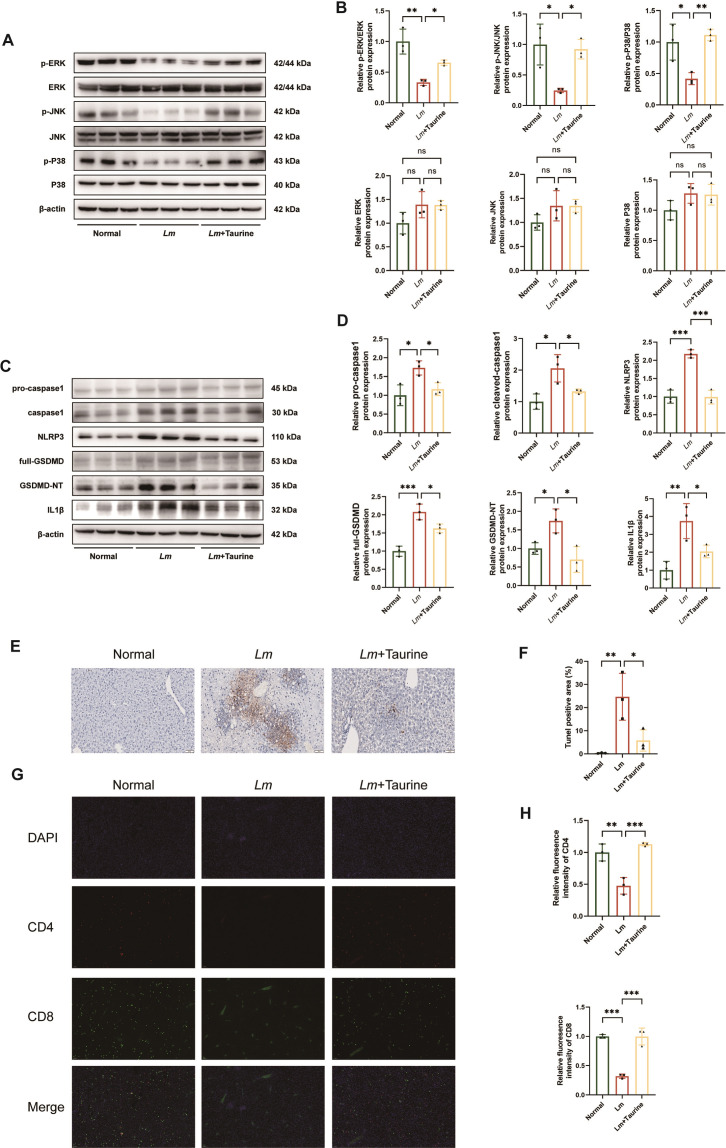
The mechanism of taurine in treating *Lm* infection. Western blotting and quantification analysis of the MAPK signaling pathway in the liver tissue (**A and B**). Western blotting and quantification analysis of the pyroptosis-related proteins in the liver tissue (**C and D**). TUNEL staining and quantification analysis of the liver (**E and F**). The immunofluorescence staining of the liver shows CD4^+^ cells in red, CD8^+^ cells in green, and cell nuclei counterstained with DAPI in blue (**G and H**). Data are expressed as mean ± SD (*n* = 3 biological replicates per group). *P* values were determined by one-way ANOVA with Tukey’s test. *P < 0.05, **P < 0.01, and ***P < 0.001, *P* > 0.05, not significant (ns).

Taurine treatment exerted dual regulatory effects on key signaling pathways related to *Lm* infection in the liver. Western blot analysis reveals that taurine elicits a cascade activation of MAPK pathway (with increased phosphorylation of p38/JNK/ERK) while simultaneously inhibiting NLRP3 inflammasome assembly, as evidenced by decreased NLRP3 protein, cleaved caspase-1, and the N-terminal fragment of gasdermin D expression (Fig. 6A through D). To establish the functional importance of p38 MAPK activation, we employed the inhibitor SB203580 *in vivo*. Pharmacological inhibition of p38 significantly attenuated the therapeutic effects of taurine, as evidenced by reduced survival rates, impaired bacterial clearance in liver and spleen, and suppressed restoration of splenic CD4 and CD8^+^ T cells (Fig. S5), indicating that p38 MAPK activation is essential for taurine’s efficacy. Immunofluorescence staining also provided direct visual evidence that taurine markedly reduced the formation of ASC specks and their co-localization with caspase-1, indicating suppression of NLRP3 inflammasome activation (Fig. S4). This regulatory pattern holds significant functional implications. Quantitative analysis via TUNEL staining confirms that taurine intervention reduces the rate of *Lm*-induced hepatocyte pyroptosis by approximately 19% (Fig. 6E and F). Immunofluorescent assays reveal that the infiltration of CD4^+^ and CD8^+^ T cells in liver tissue increases by 2.4- and 3.1-fold, respectively (Fig. 6G and H). The above results reveal the multiple regulatory mechanisms of taurine, including activation of the MAPK-dependent cell survival signaling axis, blockade of NLRP3-mediated pyroptotic execution programs, and remodeling of the antimicrobial immune microenvironment to enhance T-cell responses.

## DISCUSSION

*Lm* is a gram-positive, facultatively anaerobic intracellular parasitic bacterium belonging to the genus *Listeria*. It is an important opportunistic pathogen causing human listeriosis and the most significant foodborne pathogen of public health concern. *Lm* is highly concerning due to its environmental persistence and association with high morbidity and mortality. Current therapeutic strategies for human *Lm* infection primarily rely on antibiotics, which target bacterial survival and replication by disrupting cell wall integrity, protein synthesis, and other vital processes (21). However, with the widespread and excessive use of antibiotics, bacterial resistance and severe side effects are increasingly prevalent, underscoring the need for non-antibiotic treatments for *Lm* infections (21). Dietary interventions or natural food-derived compounds have emerged as safe and promising alternatives for managing health issues. Taurine, an abundant essential amino acid in the human body, is a natural product with diverse physiological and pharmacological activities. It was first isolated from bovine bile, hence its name. Taurine can be synthesized or extracted through various methods, including isolation from animal sources (e.g., fish and meat), microbial fermentation, and advanced biotechnological approaches involving enzymes and microorganisms (22). Over the past three decades, research on taurine’s role in metabolic syndrome has grown steadily, establishing a robust research framework (23). However, the research on it in infectious diseases has gradually become the focus only in recent years. In *Edwardsiella tarda* infection, taurine improved survival rates by modulating immune-related gene expression, thereby enhancing the host’s immune response (24). Given its established safety and low toxicity, taurine represents a viable therapeutic candidate. Our study demonstrated that taurine significantly alleviated *Lm*-induced oxidative stress and inflammation in mouse tissues, potentially via suppression of the NLRP3 pathway. Additionally, taurine activated the MAPK pathway, reversed *Lm*-mediated lymphocyte depletion, and increased activated CD8^+^ T cells, suggesting a critical role in T-cell activation.

Bacterial culture experiments have indeed confirmed that taurine can effectively inhibit the growth of *Lm*. Furthermore, this inhibitory effect was determined to be specific, as it was significantly greater than that caused by an equiosmolar mannitol control, thereby ruling out osmolarity as a confounding factor and suggesting the involvement of a more specific mechanism. Through transcriptomic analysis, we found that taurine could downregulate the expression of metabolism-related genes, thus inhibiting the growth and biofilm formation of *Lm*. Listeriolysin O (LLO), as a pore-forming protein secreted by bacteria, is an indispensable molecular weapon in *Lm* survival and infection. The presence of LLO is a hallmark of *Lm* pathogenicity, and it’s an important target for developing novel antimicrobial strategies (25–27). The hemolysis assay result, which reflects the levels of LLO, demonstrates that taurine significantly suppresses hemolytic activity, hinting at its potential role in attenuating *Lm* pathogenicity. Consistent with this, the qPCR outcomes of prokaryotic virulence gene hlyA encoding LLO further corroborated these findings (data not shown). LLO generates reactive oxygen species (ROS), affects potassium (K^+^) and calcium (Ca²^+^) ion fluxes, and impairs mitochondrial integrity, all of which ultimately lead to the assembly and activation of the NLRP3 inflammasome, which in turn activates caspase-1, promotes the maturation and release of pro-inflammatory cytokines such as IL-1β and IL-18, culminating in a vigorous inflammatory response (28–30). It has been reported that in *L. monocytogenes* mutants lacking LLO, the production of IL-1β and the activation of the NLRP3 inflammasome are impaired (31). Consistent with these findings, taurine, capable of effectively inhibiting the production of toxins, has the ability to diminish the activation of the NLRP3 and caspase-1, leading to a reduction of the pro-inflammatory cytokine IL-1β. Importantly, the combination of taurine and MCC950 did not yield an additive or synergistic inhibitory effect on pyroptosis compared to either treatment alone. This observation strongly suggests that taurine and MCC950 operate within the same molecular pathway and, specifically, that taurine exerts its antipyroptotic effects primarily through the inhibition of the NLRP3 inflammasome.

A key finding of our study is that taurine has good pharmacokinetic characteristics, which provides a compelling explanation for its effectiveness in *Lm* infection. Although the 100 mM concentration required for direct antibacterial effects *in vitro* is high, our PK data convincingly shows that a dose of 400 mg/kg can achieve and maintain millimolar levels of taurine in the target organs, the spleen and liver, throughout the entire critical period of infection. This remarkable organ specificity ensures that taurine is precisely delivered to the “front line” of *Lm* infection.

*Lm* infection could induce apoptosis, depending on a number of factors, including the bacterial inoculum size, the duration of infection, the type of host cell involved, and the state of the host immune status. Lymphocyte apoptosis is a well-recognized consequence of early *Lm* infection in mice (typically 1–3 days) and occurs in a dose-dependent manner 24 post-infection, with LLO mediating this process. The antiapoptotic effect of taurine has been described in several studies. Taurine treatment ameliorated apoptosis in diabetic nephropathy through activation of the AKT pathway, increased protein levels of Bax and Bcl-2, and attenuated the mRNA expression of cysteinylaspartate-specific protease 3 and caspase-9 (32). Similarly, taurine inhibited hippocampal neuronal apoptosis via NGF-Akt/Bad pathway and regulated mitochondria-mediated apoptosis to attenuate aflatoxin-induced liver injury (33). Our *in vitro* experiment indeed confirmed the emergence of early apoptosis, and taurine could reverse this process, establishing its protective capabilities against cell death initiated during the early phases of *Lm* infection. However, as the infection progresses and the immune system becomes increasingly activated, pyroptosis tends to become more pronounced. The cytokine storm data from the *Lm* infection model aligns with reported pyroptosis-related inflammatory factor release patterns. This correlation matches the inflammatory amplification mechanism seen in atherosclerosis models (34). Evidence is accumulating that taurine has beneficial effects on pyroptosis. Studies show that taurine can alleviate and prevent the occurrence of schistosomiasis-associated liver fibrosis by suppressing NLRP3 activation and subsequent pyroptosis (33). It can also mitigate As_2_O_3_-induced NLRP3 inflammasome cascade and pyroptosis in NASH (10). Similar to these results, our animal studies have demonstrated the occurrence of pyroptosis in mouse livers in response to *Lm* infection, and these effects could be reversed by taurine administration. Taken together, these findings suggest taurine’s antimicrobial effects may involve pyroptosis regulation.

Studies have shown that the serum taurine level in IBD patients is significantly reduced, and the level of taurine is negatively correlated with the disease activity (35). In other infection-related studies, taurine showed corresponding antioxidant and anti-inflammatory effects. Taurine inhibited NADPH oxidase activity in *Streptococcus uberis*-induced mouse mastitis and inhibited the activation of the MAPK signaling pathway, which alleviated the oxidative stress level and inflammation in mice with mastitis and served as a therapeutic effect (36). *In vitro* experiments with *Escherichia coli* and *Staphylococcus aureus* infections, taurine pretreatment inhibited the upregulation of TLR2 and TLR4 and the production of ROS to some extent and reduced the level of iNOS in cells, thereby modulating the inflammatory response during bacterial infection and preventing cellular damage by increasing the antioxidant capacity of cells (37). Not only is innate immunity essential for the initial defense against invading pathogens, but adaptive immunity also plays a critical role, particularly in achieving full clearance of *Lm*. Within the adaptive immune response, CD4^+^ T cells and CD8^+^ T cells produce IFN-γ, which activates macrophages, enhancing their bactericidal activity. Intrinsic and adaptive immunity complement each other and work together to remove pathogenic bacteria (38). We found that taurine treatment effectively restored the decrease in the number of splenic lymphocytes in mice infected with *Lm* by detecting the secretion of T cells and IFN-γ by flow cytometry. Both the total number of splenic cells and the total number of T cells in the spleen were notably elevated by the intervention of taurine, especially the IFN-γ-secreting CD8^+^ T cells. The synergistic effect of CD8^+^ T cells and reduced pro-inflammatory factors observed in this study is consistent with the previous studies. The potential inhibition and immunoregulation by taurine on TLR signal transduction may contribute to immune homeostasis restoration under various pathological conditions (39, 40).

**Fig 7:**
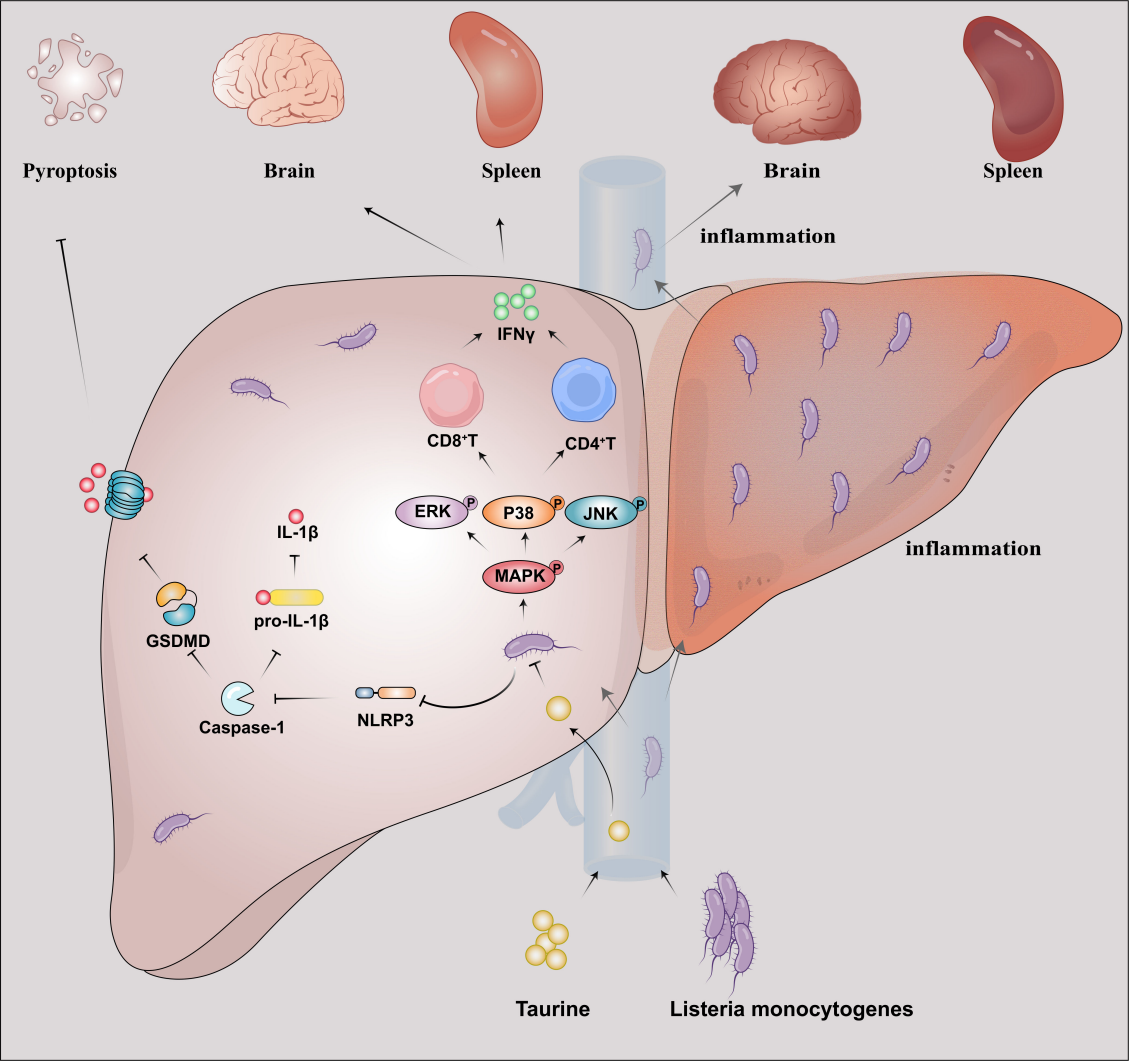
Proposed mechanism of taurine against *Lm* infection. Taurine targets *Lm* virulence and host pyroptosis. Taurine inhibits bacterial pathogenesis while balancing immune responses: activating MAPK signaling pathway for T-cell immunity and suppressing NLRP3/GSDMD-mediated pyroptosis.

The role of the MAPK pathway in cell proliferation has been widely recognized in the fields of biology and medicine. The MAPK family includes several members, such as ERK, JNK, and p38 kinase, all of which are involved in the cell’s response to external signals. When a cell receives a proliferation signal, such as those from growth factors, this signal activates the receptor tyrosine kinase located on the cell membrane. Subsequently, a series of signaling events occur that culminate in the activation of MAPKs. Activated MAPKs enter the nucleus, where they can phosphorylate and activate multiple transcription factors, which in turn influence gene expression, promoting cell cycle progression and cell proliferation (38). Our study found that taurine promotes lymphocyte proliferation through the MAPK pathway. This mechanism resembles its role in spermatogenic cell proliferation during male reproduction. These findings indicate that taurine coordinates cell proliferation in multiple physiological systems via the conserved MAPK signaling pathway (41). The abstract figure vividly depicts the therapeutic mechanism of taurine in this study (Fig. 7). Our *in vivo* inhibitor studies further solidified this mechanism, demonstrating that pharmacological blockade of p38 MAPK with SB203580 effectively reversed the benefits conferred by taurine, including survival, bacterial clearance, and T-cell recovery. This key finding establishes a causal role for p38 MAPK activation in mediating taurine’s therapeutic effects. While the MAPK pathway is often associated with pro-inflammatory responses, its role in immune cell proliferation and survival is equally critical. The seemingly paradoxical observation that MAPK activation correlates with improved outcomes in our study can be explained by its fundamental function in promoting lymphocyte expansion and fitness. The critical role of MAPK activation in facilitating CD8^+^ T-cell expansion, a process essential for effective pathogen clearance, has been elucidated in other infection models (42), thereby providing a mechanistic foundation for our observation of enhanced T-cell responses following taurine treatment. In the context of a severe bacterial infection that triggers extensive lymphocyte apoptosis, the ability of taurine to activate MAPK signaling likely provides a necessary pro-survival and pro-proliferative signal to T cells, thereby countering the *Lm*-induced lymphodepletion and restoring adaptive immunity.

As a paradigmatic model organism, *L. monocytogenes* has complex pathogenic mechanisms. Thus, our investigation targeted both the virulence and metabolism of the bacteria while simultaneously augmenting the host immune defenses to combat invaders to achieve therapeutic effects. A pivotal finding of this study is the classification of taurine as a bacteriostatic agent against *Lm*. The primary role of it is to halt bacterial replication rather than to induce lethal damage. By significantly suppressing key virulence factors, such as listeriolysin O and biofilm, and by inhibiting growth and disrupting metabolism, taurine disarms the pathogen and launches a multi-faceted assault on bacterial fitness, which inherently enhances host defense by containing infection and mitigating tissue damage. Moreover, the immunomodulatory effects of taurine greatly enhance this direct antimicrobial activity, as taurine can promote the proliferation and function of immune cells. So we propose that taurine orchestrates a cooperative defense mechanism: it simultaneously weakens the pathogen and primes the immune system for more effective clearance, ultimately culminating in the significant protection observed in our *in vivo* model. In this way, our findings not only underlie the mechanism of *Lm* infection but also provide a novel, safe, and non-toxic therapeutic strategy.

It is important to note that our study utilized an intravenous infection model, which, while not mimicking the natural oral route of infection, was selected to provide a synchronized and robust systemic challenge for the precise dissection of taurine’s immunomodulatory mechanisms in the bloodstream and peripheral organs. In future work, we will employ oral infection models to confirm the efficacy of taurine in preventing the initial bacterial translocation from the gut. Although this study demonstrates taurine’s efficacy in murine models, we acknowledge two key limitations: the translational gap between murine and human immune responses and the lack of long-term immunity data beyond acute infection phases. Future studies will address these gaps by validating taurine in humanized models and evaluating sustained immune protection. Nevertheless, this work opens up new biomedical applications for taurine, demonstrating its potential as a biotherapeutic agent in medical practice.

## Data Availability

The data that support the findings of this study are openly available in NCBI SRA at https://www.ncbi.nlm.nih.gov/sra, 6 September 2024, with accession number PRJNA1157387.

## SUPPLEMENTAL MATERIAL

Fig. S1 msystems.01043-25-s0001.tif Determination of the MIC and MBC of taurine against *Lm*.

Fig. S2 msystems.01043-25-s0002.tif Taurine downregulates the expression of virulence and metabolism-associated genes in *Lm*.

Fig. S3 msystems.01043-25-s0003.tif The protective effect of taurine against *Lm*-induced pyroptosis is dependent on NLRP3 inflammasome inhibition.

Fig. S4 msystems.01043-25-s0004.tif Taurine inhibits NLRP3 inflammasome activation in the liver.

Fig. S5 msystems.01043-25-s0005.tif Inhibition of p38 MAPK attenuates the protective effects of taurine in mice infected with *Lm*.

Fig. S6 msystems.01043-25-s0006.docx Western blotting full uncropped gel images.

Table S1 msystems.01043-25-s0007.docx Conversion of key taurine concentrations between mass per volume and molarity.

Table S2 msystems.01043-25-s0008.docx Sequence of primers.

Table S3 msystems.01043-25-s0009.docx RNA-seq sequencing data and quality control metrics.

ASM does not own the copyrights to Supplemental Material that may be linked to, or accessed through, an article. The authors have granted ASM a non-exclusive, world-wide license to publish the Supplemental Material files. Please contact the corresponding author directly for reuse.
