# Supplementary figures and images for: Taurine attenuates Listeria monocytogenes-induced inflammation and pyroptosis in mouse model by regulating MAPK and NLRP3/caspase-1/GSDMD pathways
Source: mSystems. 2026 Feb 2;11(3):e01043-25. doi: 10.1128/msystems.01043-25 (PMC13011350; doi:10.1128/msystems.01043-25)

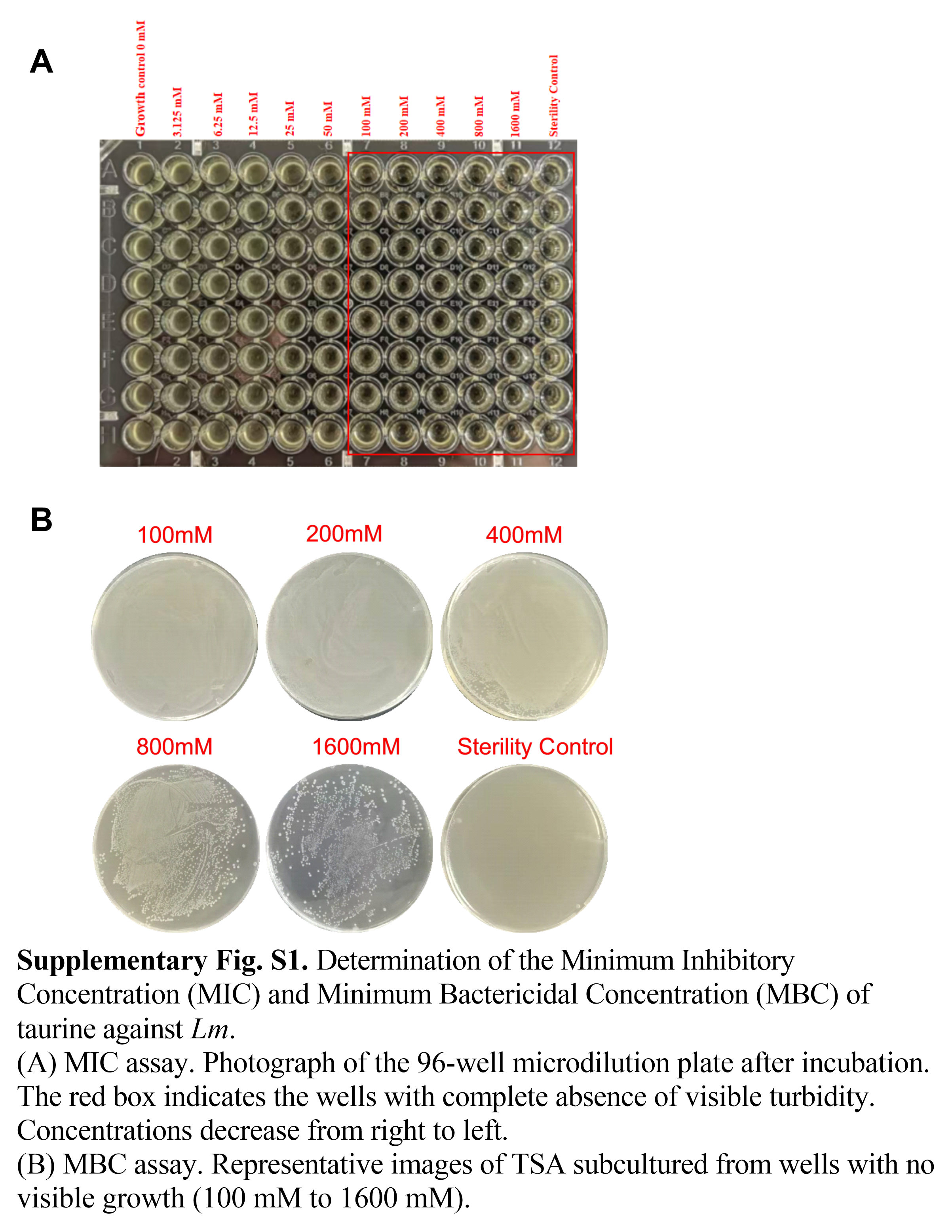

Supplement: Fig. S1 — Determination of the MIC and MBC of taurine against Lm. [file msystems.01043-25-s0001.tif]

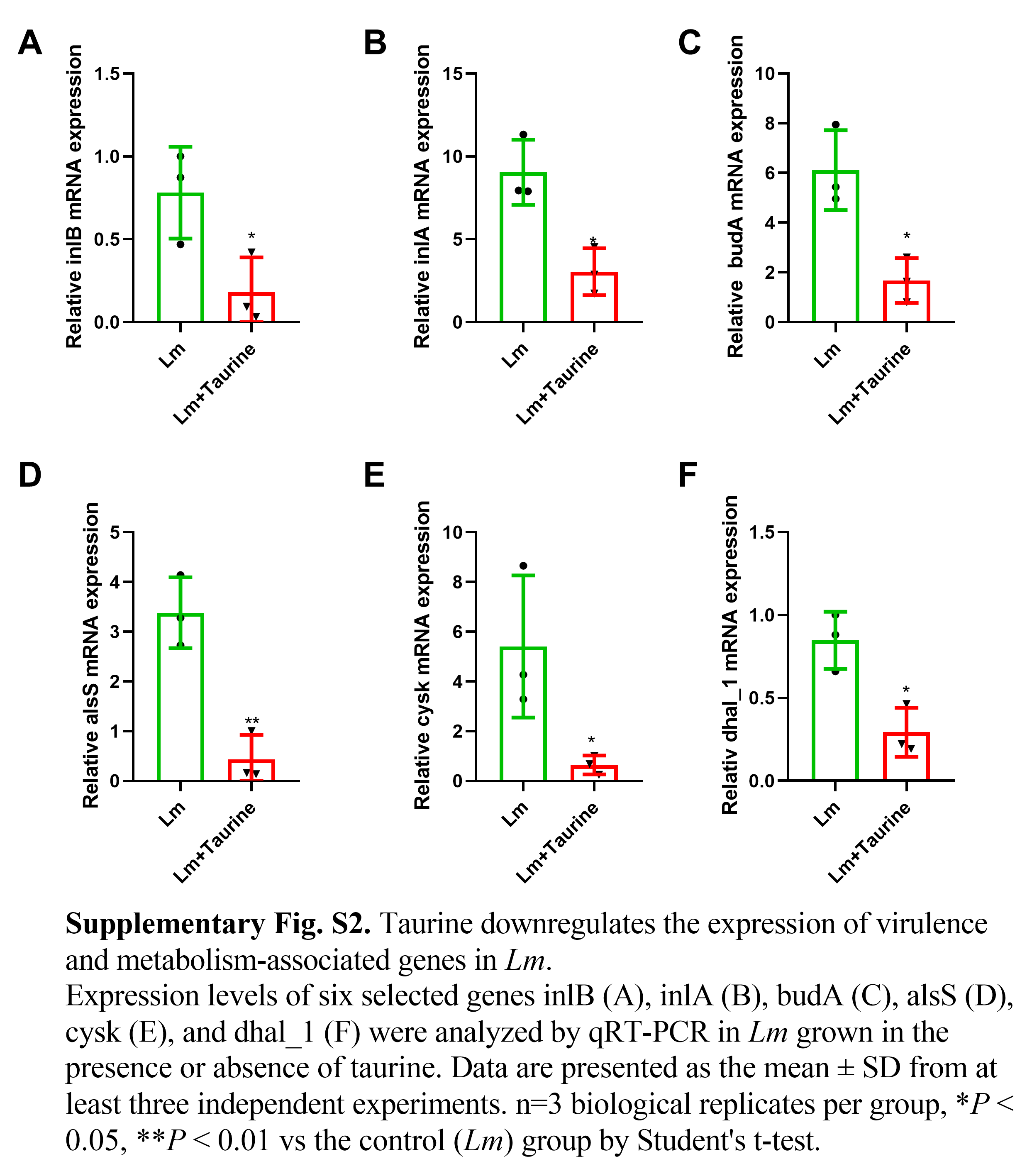

Supplement: Fig. S2 — Taurine downregulates the expression of virulence and metabolism-associated genes in Lm. [file msystems.01043-25-s0002.tif]

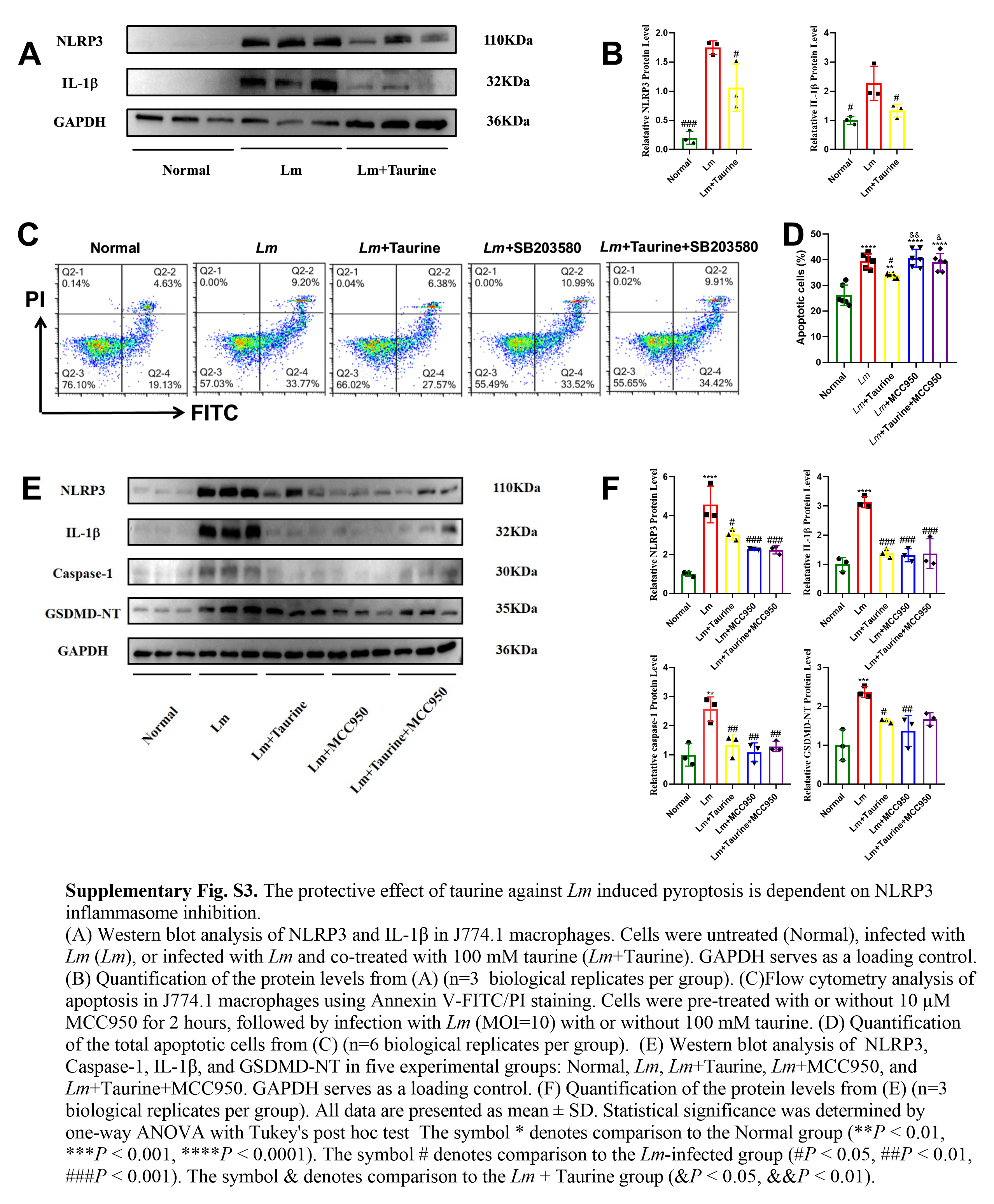

Supplement: Fig. S3 — The protective effect of taurine against Lm-induced pyroptosis is dependent on NLRP3 inflammasome inhibition. [file msystems.01043-25-s0003.tif]

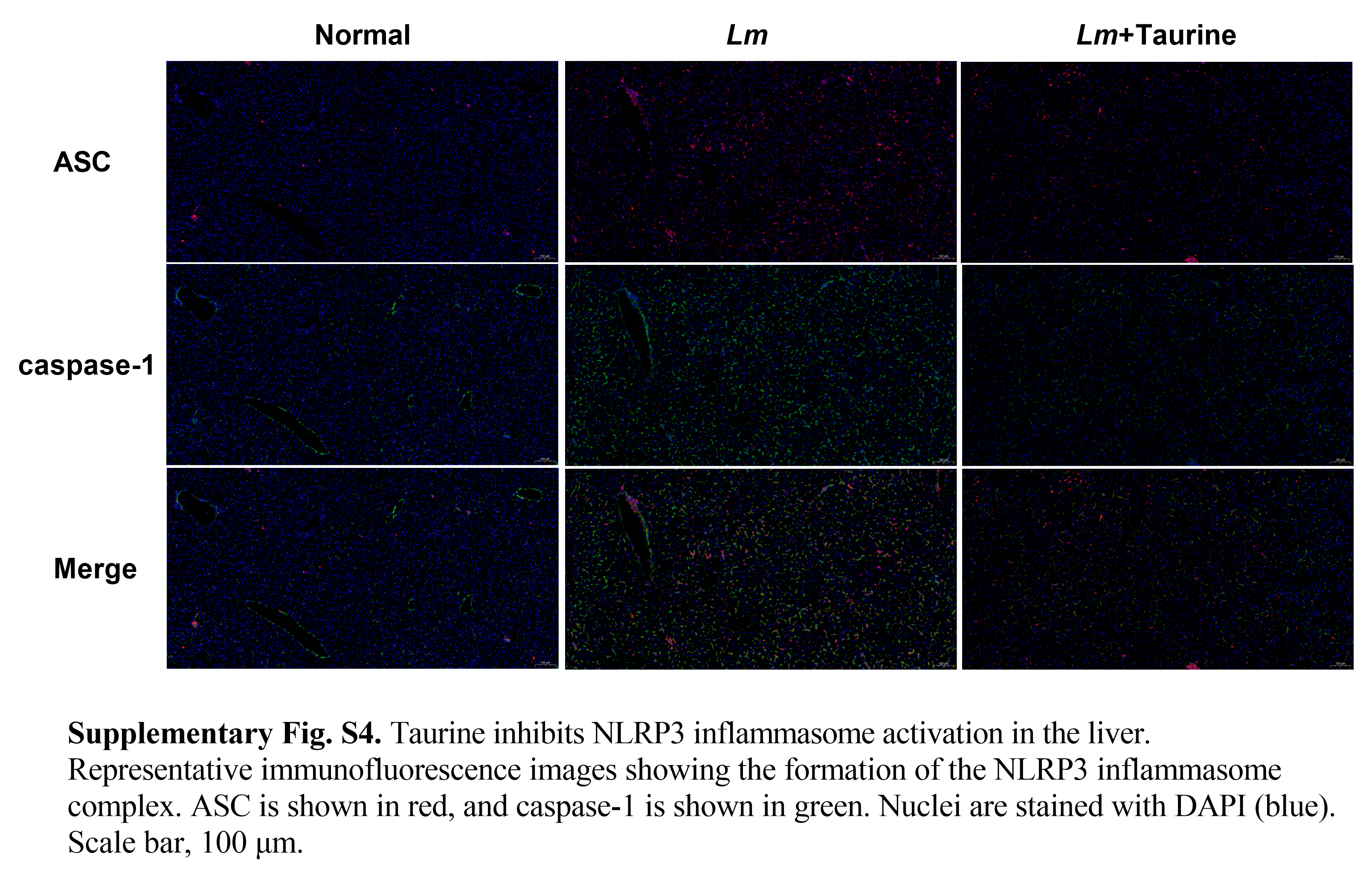

Supplement: Fig. S4 — Taurine inhibits NLRP3 inflammasome activation in the liver. [file msystems.01043-25-s0004.tif]

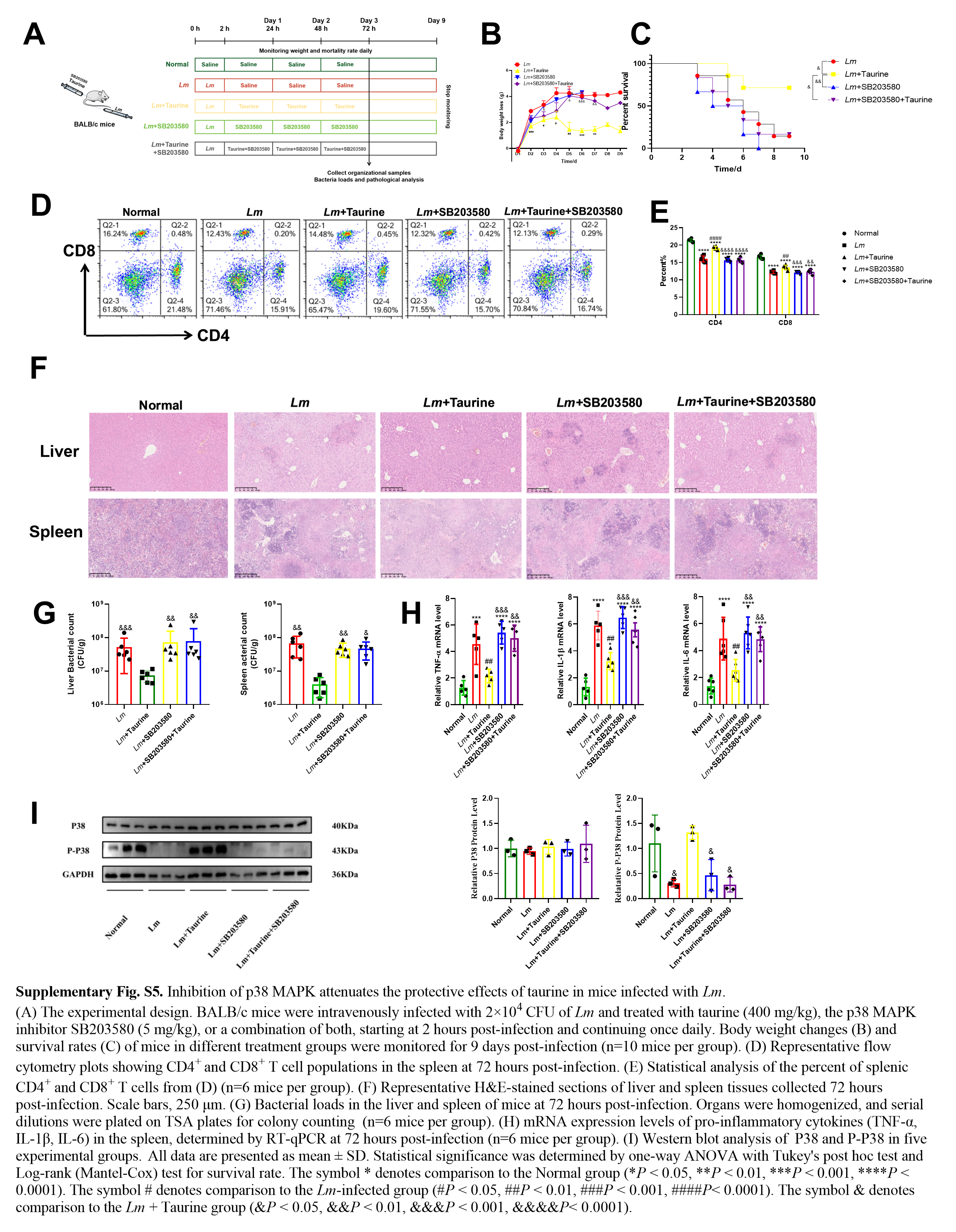

Supplement: Fig. S5 — Inhibition of p38 MAPK attenuates the protective effects of taurine in mice infected with Lm. [file msystems.01043-25-s0005.tif]
