# Supplementary material for: Taurine attenuates Listeria monocytogenes-induced inflammation and pyroptosis in mouse model by regulating MAPK and NLRP3/caspase-1/GSDMD pathways
Source: mSystems. 2026 Feb 2;11(3):e01043-25. doi: 10.1128/msystems.01043-25 (PMC13011350; doi:10.1128/msystems.01043-25)
Supplement: Fig. S6 — Western blotting full uncropped gel images. [file msystems.01043-25-s0006.docx]

**Supplementary Fig. S6**

**A.**


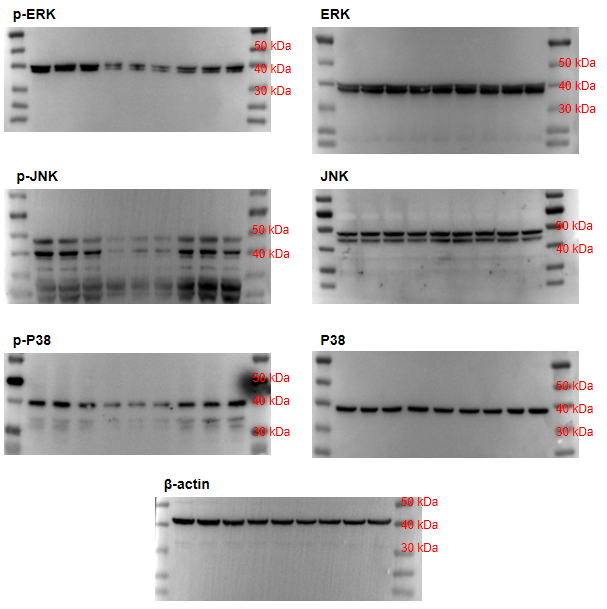


**B.**


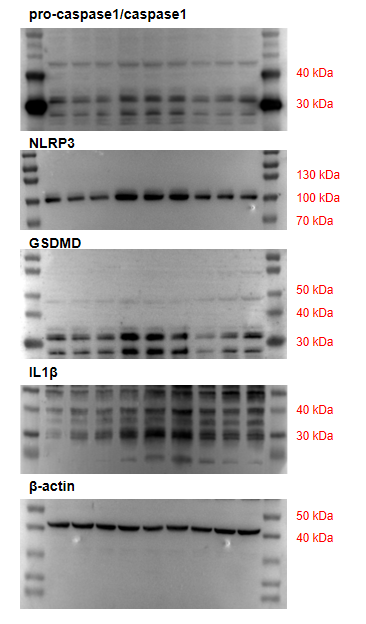


**C.**


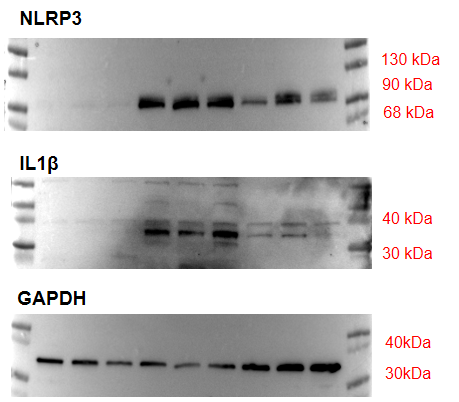


**D.**


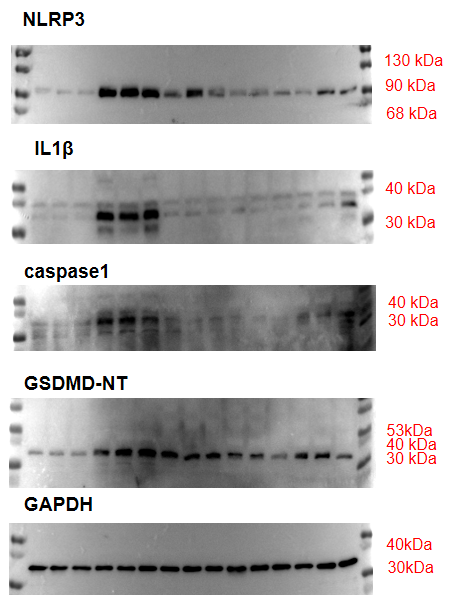


**E.**


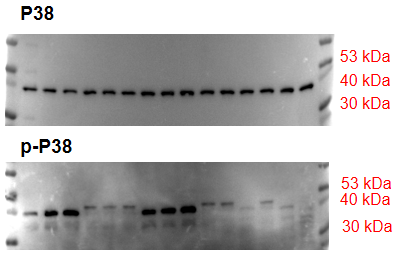


**Supplementary Fig. S6 (**A) Western blotting full uncropped gel images of the MAPK signaling pathway in the liver tissue. (B) Western blotting full uncropped gel images of the pyroptosis related proteins in the liver tissue. (C) Western blotting full uncropped gel images of the pyroptosis related proteins in the cell J774.1. (D) Western blotting full uncropped gel images of the pyroptosis related proteins in the cell J774.1 treated with MCC950. (E) Western blotting full uncropped gel images of the P38/P-P38 in the liver tissue.
