# Supplementary material for: Taurine attenuates Listeria monocytogenes-induced inflammation and pyroptosis in mouse model by regulating MAPK and NLRP3/caspase-1/GSDMD pathways
Source: mSystems. 2026 Feb 2;11(3):e01043-25. doi: 10.1128/msystems.01043-25 (PMC13011350; doi:10.1128/msystems.01043-25)
Supplement: Table S1 — Conversion of key taurine concentrations between mass per volume and molarity. [file msystems.01043-25-s0007.docx]

**Supplementary Table S1**

Conversion of key taurine concentrations between mass per volume (mg/mL) and molarity (mM)

| Application/ Experiment | Concentration (mg/mL) | Equivalent Concentration (mM) |
| --- | --- | --- |
| In vitro: Bacterial culture | 0.01 | 80 |
|  | 0.05 | 400 |
|  | 0.10 | 800 |
| In vitro: J774.1 cells |  | 100 |
| In vivo: Mouse model | 100 mg/kg |  |
|  | 200 mg/kg |  |
|  | 400 mg/kg |  |
|  | 800 mg/kg |  |

Calculations are based on the molecular weight of taurine (125.15g/mol). Concentrations used in both in vitro and in vivo experiments are provided.
