# Supplementary material for: Taurine attenuates Listeria monocytogenes-induced inflammation and pyroptosis in mouse model by regulating MAPK and NLRP3/caspase-1/GSDMD pathways
Source: mSystems. 2026 Feb 2;11(3):e01043-25. doi: 10.1128/msystems.01043-25 (PMC13011350; doi:10.1128/msystems.01043-25)
Supplement: Table S2 — Sequence of primers. [file msystems.01043-25-s0008.docx]

**Supplementary Table S2**

Sequence of primers

| Gene | |  | | Primers |
| --- | --- | --- | --- | --- |
| β-actin | Forward | | 5'GATTACTGCTCTGGCTCCTAGC3' | |
|  | Reverse | | 5'GACTCATCGTACTCCTGCTTGC3' | |
| IL-1β | Forward | | 5'CTTCAGGCAGGCAGTATCAC3' | |
|  | Reverse | | 5'TCACACACCAGCAGGTTATC3' | |
| IL-6 | Forward | | 5'AACCTTCCAAAGATGGCTGAA3' | |
|  | Reverse | | 5'CAGGAACTGGATCAGGACTTT3' | |
| TNF-α | Forward | | 5' CCCTCACACTCAGATCATCTTC3' | |
|  | Reverse | | 5' GTTGGTTGTCTTTGAGATCCAT3' | |
| iNOS | Forward | | 5'TGGCTGTGCTCCATAGTTT3' | |
|  | Reverse | | 5'GACCAGCCAAATCCAGTCT3' | |
| 16s | Forward | | 5'TTAGCTAGTTGGTAGGGT3' | |
|  | Reverse | | 5'AATCCGGACAACGCTTGC3' | |
| inlB | Forward | | 5'TATGCAGCATGGCTTGTAACC3' | |
|  | Reverse | | 5'GTTCTTGCAGAGATGGCACG3' | |
| inlA | Forward | | 5'TGGCTTTCAGCTGGGCATAA3' | |
|  | Reverse | | 5'AGCTGGTGCAATTAAAGCGC3' | |
| budA | Forward | | 5'TGGCAAAGCTGGAACACTTT3' | |
|  | Reverse | | 5'TGCGGTATTGCCTCTTTTGT3' | |
| alsS | Forward | | 5'ACTAGGTGTACAAGTGGCGC3' | |
|  | Reverse | | 5'ACGCCGTCTGCTTTTCAGTA3' | |
| cysk | Forward | | 5'AGGCGGAACAGTAACTGGTG3' | |
|  | Reverse | | 5'CGATGGAGAACCACCGCTAA3' | |
| dhal_1 | Forward | | 5'CGCCACCAACTTTTCCAAGG3' | |
|  | Reverse | | 5'AACCGGACTGGACTCGGATA3' | |
