# Supplementary material for: Taurine attenuates Listeria monocytogenes-induced inflammation and pyroptosis in mouse model by regulating MAPK and NLRP3/caspase-1/GSDMD pathways
Source: mSystems. 2026 Feb 2;11(3):e01043-25. doi: 10.1128/msystems.01043-25 (PMC13011350; doi:10.1128/msystems.01043-25)
Supplement: Table S3 — RNA-seq sequencing data and quality control metrics. [file msystems.01043-25-s0009.docx]

**Supplementary Table S3**

RNA-seq sequencing data and quality control metrics.

| Sample name | Raw reads | Clean reads | Clean bases | Q20(%) | Q30(%) | GC content(%) |
| --- | --- | --- | --- | --- | --- | --- |
| Lm1 | 7069088 | 6909448 | 1.0G | 97.56 | 92.82 | 39.16 |
| Lm2 | 7162448 | 7033002 | 1.1G | 97.66 | 93.03 | 39.2 |
| Lm3 | 7557694 | 7389436 | 1.1G | 97.65 | 93.14 | 39.17 |
| Lm_Taurine1 | 7765316 | 7587834 | 1.1G | 97.71 | 93.18 | 39.25 |
| Lm_Taurine2 | 7676970 | 7495632 | 1.1G | 97.58 | 92.85 | 39.47 |
| Lm_Taurine3 | 7837152 | 7674106 | 1.2G | 97.61 | 93.04 | 39.34 |
